# How stakeholder engagement influenced a randomized comparative effectiveness trial testing two Diabetes Prevention Program interventions in a Marshallese Pacific Islander Community

**DOI:** 10.1186/s12967-019-1793-7

**Published:** 2019-02-11

**Authors:** Pearl A. McElfish, Britni L. Ayers, Holly C. Felix, Christopher R. Long, Zoran Bursac, Joseph Keawe‘aimoku Kaholokula, Sheldon Riklon, Williamina Bing, Anita Iban, Karen Hye-cheon Kim Yeary

**Affiliations:** 1 University of Arkansas for Medical Sciences, Northwest Campus, 1125 North College Ave, Fayetteville, AR 72703 USA; 2 Florida International University, 11200 SW 8th St., AMHC5-463, Miami, FL 33199 USA; 3 University of Hawaii at Manoa, 677 Ala Moana Blvd. 1016, Honolulu, HI 96813 USA; 4 Springdale School District, Springdale, AR USA; 5 University of Arkansas for Medical Sciences, 4301 W. Markham St., Little Rock, AR 72205 USA

**Keywords:** PCOR, CBPR, Pacific Islander, Marshallese, Diabetes Prevention Program, Type 2 diabetes, RCT

## Abstract

**Background:**

Marshallese face significant health disparities, with particularly high rates of type 2 diabetes. Engaging stakeholders in the research process is essential to reduce health inequities.

**Methods:**

A community- and patient-engaged research approach was used to involve community Marshallese stakeholders in a randomized comparative effectiveness trial testing two Diabetes Prevention Program interventions.

**Results:**

The article outlines the engagement process and the specific influence that stakeholders had on the research planning and implementation, discussing the areas of agreement and disagreement between community and patient stakeholders and academic investigators and documenting changes to the research protocol.

**Conclusion:**

The article provides an example of methods that can be used to design and conduct a randomized controlled trial testing with a population who has been underrepresented in research and suffered significant historical trauma.

## Background

Pacific Islanders are one of the fastest growing populations in the United States (US), with a 40% increase from 2000 to 2010 [1]. Southern and Midwestern states, such as Arkansas, Kansas, Missouri, and Oklahoma, had particularly rapid growth in Pacific Islander communities [1]. Most of the Pacific Islander population growth in these states are Micronesian populations from the Compact of Free Association (COFA) nations, including Marshallese from the Republic of the Marshall Islands (RMI). As part of the COFA, Marshallese can freely migrate to the US [2–4]. The Marshallese began migrating to Southern and Midwestern states for work and educational opportunities [5]. Arkansas now has the largest population of Marshallese in the continental US [6–9], and rapid growth in the Marshallese population continues in Arkansas, Kansas, Missouri, and Oklahoma [9].

The US has a complex and contentious history with the Marshallese community. Between 1946 and 1958 the US military tested nuclear weapons on the RMI, which were equivalent to more than 7000 Hiroshima-sized bombs [10, 11]. While the Marshallese who lived on the bombed islands and atolls were relocated, Marshallese living on nearby atolls were not. The nuclear testing contaminated food and water supplies and disrupted the Marshallese traditional way of life, which included self-reliance on fish and local fruits and vegetables. These traditional foods were replaced with commodities high in fat, refined carbohydrates, and sodium negatively affecting subsequent generations of Marshallese [12].

The US nuclear testing program exposed Marshallese to significant levels of nuclear radiation [10, 11, 13–22]. After the nuclear weapons testing, US scientists set up a study called Project 4.1 to better understand the effects of nuclear radiation on humans [10]. Marshallese who had been exposed to direct nuclear fallout were brought to Kwajalein Atoll for examination as part of Project 4.1. The research was conducted without translation of the study information into Marshallese and without informed consent [10]. The nuclear testing and subsequent research of Project 4.1 perpetuated historical trauma evidenced by Marshallese community members’ deep mistrust of research and health care providers that are past down to the next generation [10, 23, 24]. Culturally-insensitive researchers and providers only further serve to exacerbate their trauma; thus, leading to health care access issues for Marshallese. Because of the historical trauma perpetuated by the US nuclear weapons testing program and Project 4.1, many Marshallese are skeptical of health care providers and reluctant to participate in research. One way to address health disparities and historical trauma is through community- and patient-engaged research.

The Patient-Centered Outcomes Research Institute (PCORI) was established to fund patient-centered outcomes research (PCOR) that evaluates research questions and meaningful outcomes to patients and caregivers [25]. PCORI posits that incorporating the patient perspective into health care research enhances usefulness and expedites the uptake of research into practice. PCOR is predicated on community-engaged research principles as it seeks to involve patients and community stakeholders in all areas of the research process. Community- and patient-engaged research has demonstrated effectiveness among underserved and disparate populations who are often underrepresented in research [26–30].

In 2012, the authors began working with the Marshallese to better understand the health disparities present in this population using a community- and patient-engaged approach to conduct qualitative and quantitative needs assessments [31–35]. Articles describing the process and results of this engagement are published elsewhere [35, 36]. Needs assessment data revealed rates of type 2 diabetes (38%), prediabetes (33%), hypertension (41%), and overweight/obesity (90%) that are substantially higher among the Marshallese than the general US population [34]. Prevention of type 2 diabetes was prioritized as the top health concern and risk by the Marshallese community in Arkansas [37, 38]. This article describes the process of developing a randomized controlled trial (RCT) to compare the effectiveness of two Diabetes Prevention Programs using community- and patient-engaged research principles with Marshallese stakeholders’ input.

## Methods

To address type 2 diabetes, a diverse community-academic research team was required. The team included: three Marshallese community co-investigators, four academic investigators with expertise in engaged research, a clinical pharmacist who provided type 2 diabetes education, two nurse health educators, a family physician, a biostatistician with expertise in comparative effectiveness research, and two endocrinologists. The research was also supported by two stakeholder advisory boards: a Marshallese faith-based organization (FBO) stakeholder advisory board with 21 members and a broader community stakeholder advisory board with 11 Marshallese community members.

Between June 2015 and December 2017, the community-academic research team engaged in discussions with Marshallese stakeholder advisory boards regarding all aspects of the research design. The information for this article was derived from the process notes. The present article documents the specific influence that Marshallese stakeholders had on the research planning and implementation of a randomized comparative effectiveness trial, and discusses the areas of agreement and disagreement between Marshallese stakeholders and the research team (summarized in Table 1). The research project was funded by PCORI in 2017, and the study is currently being conducted.

**Table 1:** Stakeholder engagement in all phases of the study

Area	Elements influenced through stakeholder involvement
Establish need and formulating research question	Marshallese stakeholders prioritized prevention of type 2 diabetes as the top health concern and risk in the community
Choosing comparator interventions	The two interventions/comparators (The WORD DPP and PILI ‘Ohana DPP) were chosen because they met the Marshallese stakeholders criteria: engaged family and friends, incorporated faith and FBO leaders, included cultural adaptations
Design	Marshallese stakeholders initially wanted participants and FBO to choose which intervention they receive. In-depth discussion was needed to explain the need for random assignment
Unit of randomization	Marshallese stakeholders input helped identify importance of randomizing at the FBO level rather than the participant level to reduce contamination
Outcomes of importance, instruments and measures	Marshallese stakeholders and the research team agreed that percent body weight change from baseline to 6 months was the primary outcome. Self-reported measures for behavioral changes and family support were chosen based upon Marshallese stakeholders’ input Based upon Marshallese stakeholders input, scales for many instruments were adapted and refined to maximize construct validity among Marshallese participants. The total number of items was reduced by 36%
Recruitment	Based upon Marshallese stakeholders input, multiple recruitment methods were used to supplement FBO recruitment, including clinical referrals and social media
Data collection intervals, remuneration, and retention	Marshallese stakeholders believed that pre/post test data collection was sufficient; significant education was needed to gain agreement on 12 month post-intervention data collection. Marshallese stakeholders developed a draft retention plan that includes the utilization of a case management approach that incorporated both Marshallese research staff and Marshallese faith-based liaisons. Based upon stakeholder input, remuneration amounts increase as the study progresses to incentivize retention in the study, and closed Facebook groups were set up to allow participants to stay connected to study staff
Staffing and resource sharing	Marshallese stakeholders determined that Marshallese bilingual and bicultural research staff at the university should be responsible for recruitment, data collection, teaching interventions’ educational sessions, and study coordination. The university contracted with community based organizations to provide translation services, assist with recruitment, and facilitate dissemination. In addition, seven FBO liaisons were hired to assist with recruitment, retention, and intervention implementation. Marshallese stakeholders determined the appropriate compensation for community co-investigators, the two stakeholder advisory boards, and participants
Language	All written materials, including consent and educational materials are provided in both English and Marshallese. All stakeholder meetings and intervention sessions are facilitated in Marshallese
Dissemination	Marshallese stakeholders articulated that updates on the study’s progress (recruitment, enrollment, study progress, etc.) was as important as dissemination of the final results. Study updates are provided to the two advisory boards quarterly. In addition, broader community town hall meetings are to be held biannually to provide study updates

*FBO* Faith Based Organization, *DPP* Diabetes Prevention Program, *PILI ‘Ohana DPP* Pacific culturally adapted Diabetes Prevention Program, *WORD DPP* Wholeness, Oneness, Righteousness, Deliverance Faith-based Diabetes Prevention Program

## Process and results

### Establishing need and formulating the research question

The research question was formulated through the Marshallese stakeholders’ prioritization of prevention of type 2 diabetes as the top health concern and risk in the community [38]. Marshallese stakeholders requested that in addition to working to understand how to best treat patients with type 2 diabetes, how to best prevent type 2 diabetes was a greater priority in the Marshallese community. The research question was first articulated as “how can we prevent type 2 diabetes in the Marshallese community?” After conducting a review of evidence-based diabetes prevention interventions, and selection of the Diabetes Prevention Program (DPP) for adaptation, the research question was later refined to “which DPP program will work best in the Marshallese community?”.

### Choosing comparator interventions

The DPP has been widely translated for diverse populations [39] and the research team in collaboration with community and patient stakeholders undertook a process to identify which adapted DPP programs would be the most appropriate for Marshallese. Marshallese stakeholders discussed the elements that would be most important in the selection of an adapted DPP, including the engagement of family and friends, incorporation of faith, and involvement of FBO leaders. Stakeholders were also interested in testing an intervention that included Pacific cultural adaptations. Marshallese stakeholders considered previous, evidenced-based, DPP interventions that had been implemented in community settings. The goal was to find two interventions that stakeholders felt had the most potential for success in the Marshallese community. After reviewing several DPP interventions, two were chosen: The WORD (Wholeness, Oneness, Righteousness, and Deliverance) DPP and PILI ‘Ohana DPP. Both interventions’ core curricula incorporated all components of the DPP and emphasized increasing physical activity, eating healthy, and self-monitoring. The WORD DPP is a faith-based DPP curriculum that teaches participants to connect faith and health to meet the project’s behavioral goals. The WORD was originally developed and tested in African American FBOs using a community-based participatory approach [40, 41]. The WORD DPP was chosen because of its emphasis on faith, which Marshallese stakeholders believed would be the most appropriate for the community. PILI in the Hawaiian language means “to be close to or together” and ‘Ohana means “family”; thus, referring to the engagement of a person’s social support of friends and family to support lifestyle changes. Originally tested among Native Hawaiian and other Pacific Islander communities in Hawaii, PILI ‘Ohana DPP uses examples relevant to Pacific Islanders [42–44]. Both the WORD and PILI ‘Ohana DPP interventions have effectively reduced participant’s percentage of body weight, which is one of the primary mechanisms for diabetes risk reduction [43, 44]. Stakeholders believed the two selected comparators (The WORD DPP and PILI ‘Ohana DPP) held the most promise for success in the Marshallese community.

### Study design

The use of randomization for assignment to intervention groups was initially a point of contention. The research team believed that random assignment was necessary to maintain the study design’s rigor and meet PCORI Methodology Standards. Some stakeholders believed it would be best to allow participants or FBOs to choose the intervention in which they took part and argued that this would allow for greater participation, and retention. The research team and the other stakeholders collaboratively discussed the strengths and challenges of random assignment. All stakeholders ultimately agreed on random assignment; however, this was a point of robust discussion before a decision was reached. This discussion also guided the development of the recruitment flyer and recruitment presentation (i.e., PowerPoint slides) to better explain random assignment.

### Unit of randomization

The research team originally conceived implementing the study with participants as the unit of randomization. However, Marshallese stakeholders provided feedback that this would be very confusing for participants who went to the same FBO and knew each other well. The research team agreed that randomizing at the participant level might also increase contamination between groups. Therefore, FBOs were chosen as the unit of randomization. The fact that the research team changed the research design to match the Marshallese stakeholders’ recommendations—exemplifies shared-decision making and helped to build trust and increase the stakeholders’ confidence in providing recommendations on the research design.

### Outcomes of importance, instruments and measures

Marshallese stakeholders voiced that the most important outcomes of the study were participants: (1) understanding what they needed to do to prevent type 2 diabetes (increase in knowledge and self-efficacy), (2) making behavioral changes based upon increased knowledge and self-efficacy, (3) being able to make changes within the context of their family and community (family/community support), and (4) losing weight (including causing changes in biometric measures associated with weight loss). The research team recommended that weight loss from baseline be the primary outcome measure for power analysis and sample size determination because percentage of weight loss is the primary outcome variable for other DPP studies [45, 46] and Marshallese stakeholders agreed. Respecting the community’s preferences, the research team agreed to focus other measures on diabetes knowledge and self-efficacy, improvements in behavioral and other biometric measures, and family/community support as secondary outcomes. To capture and evaluate other outcomes, research advocated that validated instruments be used.

The research team and Marshallese stakeholders reviewed the instruments used in both The WORD and the PILI ‘Ohana DPP interventions, which incorporated validated instruments from Behavioral Risk Factor Surveillance System, the National Health and Nutrition Examination Survey, and other sources [36, 40]. Marshallese stakeholders and the research team evaluated those instruments for their appropriateness to collect the selected outcome measures. Instrument selection required several hours of discussion and debate.

The survey questions were pilot tested with Marshallese stakeholders, who subsequently advocated for two major changes. The first was reducing the response option of Likert-type scales from five to three. The second was reducing the overall number of questions by approximately 36% so that only the most relevant questions were asked. Both of these changes created significant concern for the research team. In terms of the scale change, the research team noted that reducing the scale options would drastically reduce the instruments’ ability to detect change and variability. However, Marshallese community members and the community co-investigators voiced the concern that many Marshallese participants were not accustomed to responding to Likert-type scales and would be unlikely to focus on subtle gradations between each response option; for this reason, they suggested abbreviating survey responses to no more than three options.

While some research team members did not agree with Marshallese stakeholders, the research team conceded that the primary outcome of weight loss and other biometric measures would be sufficient for a robust analysis. Thus, the final instrument used an adapted three point Likert-type scale rather than a five point scale. To address stakeholder concerns about survey length, the number of questions included in the survey was reduced by 36%. The instrument was then pilot tested again and Marshallese stakeholders confirmed the assessment prior to study launch. The final instrument includes 43 survey items and six biometric measures [47].

### Recruitment

Based upon the change to FBOs as the unit of randomization, it was critical to get the full involvement of FBOs at the beginning of the participant recruitment process. To do this, Marshallese research staff met with FBO leaders to explain the study and get their agreement to have their FBO participate in the study and accept random assignment to either intervention. FBO leaders included pastors, madam pastors, deacons, and deaconesses. After garnering FBO leaders’ support, Marshallese research staff held informational sessions within each FBO to answer any questions from the congregation.

Marshallese stakeholders recommended the use of multiple recruitment methods for participants from the FBO’s. In addition to recruiting participants from the congregations of recruited FBOs, Marshallese stakeholders suggested that clinical referrals and social media announcements be used to recruit who provided broader representation of the Marshallese community. Eligibility screening events were then held at participating FBOs. Recruitment materials were in Marshallese and English.

### Data collection intervals, remuneration and retention

Community and academic partners also discussed data collection intervals. Marshallese stakeholders believed that pre/post-test data collection was sufficient and questioned the need to extend data collection to 12 months. The research team communicated the importance of collecting maintenance data in behavioral interventions; however, Marshallese stakeholders continued to voice concerns about the feasibility and participant burden retaining participants through 12 months. To include maintenance assessment and mitigate the stakeholders’ concerns regarding retention, community and academic partners formulated a retention plan that consisted of a case management approach incorporating both Marshallese research staff and FBO liaisons (discussed in “Staffing and resource sharing”). In addition, a remuneration plan was implemented so that remuneration amounts increased for each data collection event. Based directly on Marshallese stakeholders’ input, participants will receive $20 for the first (pre-intervention) data collection, $30 for the second (immediate post-intervention; 6 months) data collection, and $40 for the third (maintenance; 12 months) data collection. In addition, stakeholders recommended that participants be continually engaged in the study through social media.

To incorporate Marshallese stakeholders’ recommendations for using social media and to meet institutional review board requirements, the research team created a closed Facebook group for each group class. While Marshallese stakeholders believed retaining and tracking Marshallese participants for the duration of the study would be challenging, the revised retention plan reflected intensive stakeholders input to maximize participant retention.

### Staffing and resource sharing

The partnership had extensive discussions concerning staffing and resource sharing. Marshallese stakeholders communicated that those implementing the study should: (1) be members of the Marshallese community, (2) bilingual (English and Marshallese), (3) have extensive health education training and experience, and (4) be fully dedicated to the research project. The community-academic research team decided that Marshallese community members would be employed by the university as health education and research staff to ensure adherence to regulations governing human-subjects’ protection and data security and to ensure primary dedication to the research project. These employees would have access to university employee benefits, which includes a tuition discount for employees and their families and a generous retirement match. Marshallese stakeholders also believed employment by the university would allow the Marshallese community access to the university’s social networks and capital important to the research employees as well as the community at large.

In addition to the seven Marshallese community members employed by the university, two contracts were provided to Marshallese community based organizations to translate study materials, provide input on study activities, and coordinate community updates and dissemination of results through town hall meetings. Marshallese stakeholders strongly recommended that the study include FBO liaisons to help facilitate recruitment and retention. Therefore, the university executed seven contracts with individuals to serve as community liaisons responsible for working with the Marshallese community-based organization and the university employed research staff to fulfill the research objectives in a culturally appropriate way. Marshallese stakeholders determined the appropriate compensation for community co-investigators, the two stakeholder advisory board members, and participants.

### Language

Based upon stakeholder input, both DPP interventions are conducted in Marshallese. Consent is conducted in either Marshallese or English based upon each participant’s language of choice. Study updates, stakeholder advisory board meetings, and dissemination town hall meetings are facilitated in Marshallese; Stakeholders reinforced that the use of Marshallese language is important to help the Marshallese community feel comfortable. It also shifts the power to the community and allows them to discuss elements and then provide only the information they want shared with the research team in English. All written materials, including consent and educational materials, are provided in both English and Marshallese.

### Dissemination

While the research is in the early stages, a dissemination plan has been drafted and intermediate dissemination has begun. Marshallese stakeholders considered updates on the study’s progress including recruitment, enrollment, and retention to be as important as dissemination of the final results. As a result, the dissemination plan includes study updates to two stakeholder advisory boards on a quarterly basis, and two annual town hall meetings with the broader community. The lead investigator welcomes the group and then Marshallese Community Co-Investigators and Marshallese staff facilitate the meeting. The lead investigator is available at the meeting to answer questions. To prevent contamination, intermediate results are not shared. After the study is completed, the results will be disseminated through an infographic in both English and Marshallese that is developed with input from the stakeholder advisory boards. The infographic will be provided to every participant, distributed to social media, and provided at a town hall meeting. No disseminated information will identify specific participants.

### Final review of the protocol

In addition to collaborating with the research team to make decisions regarding the study’s research design, evaluative outcomes, and dissemination plan, Marshallese stakeholders pilot tested the intervention through serving as mock participants and providing constructive feedback to study materials. The research team developed a study protocol that detailed the study’s methods and standard operating procedures. An iterative process for protocol review was then used whereby stakeholders and the research team introduced modifications before agreeing on the final protocol version and the study was launched.

### Stakeholder Advisory Boards continued involvement and community co-investigators

Two Marshallese stakeholder advisory boards continue to provide input to the research team through quarterly meetings. The Marshallese stakeholders were selected by the broader group to serve as Marshallese community co-investigators serve roles similar to other university investigators and provide input at monthly research team meetings. The Marshallese community co-investigators and Marshallese stakeholder advisory boards ensure the Marshallese community’s input and priorities are maintained throughout the research process.

## Discussion

Designing rigorous studies using engaged research approaches is both beneficial and challenging. Community and patient stakeholders often lack scientific methods experience and are reluctant to give input on research design. Academic researchers often find it challenging to balance the need for rigorous research design with the desires of community and patient stakeholders. Despite these challenges, stakeholder input in research design is imperative to the implementation and dissemination of research in community-based settings. Stakeholder input also increases the cultural and community-context relevance of the intervention, which can expedite the dissemination process. Much of the prior literature has outlined the principals and process for PCOR, and some articles have compared PCOR to community-based participatory research (CBPR) [30]. However, few articles have provided specific details regarding the role of stakeholder engagement in research protocols. This paper adds to the literature by describing the decision-making process of one partnership dedicated to designing, implementing, evaluating, and disseminating a trial to prevent type 2 diabetes among the Marshallese.

This research provides several lessons learned that can be integrated into other research. Using a community- and patient-engaged process required substantial time and an openness by all partners. Formulating the research protocol required an openness to divergent points of view, flexibility, patience in listening, and a willingness to change. Partners came to an easy consensus regarding the research protocol (e.g. recruitment strategies, staffing, primary outcomes), whereas other decisions came after several discussions, education of the research team and the stakeholders, and compromise (e.g. study design, unit of randomization, survey questions). It was not uncommon for further discussions to arise after initial decisions had been made. It was imperative that researchers maintained a willingness to continue dialogue and renegotiate decisions in order maintain a strong partnership and shared ownership of the research process. In this study, the additional post hoc input from stakeholders strengthened the scientific rigor of the study by changing the unit of randomization. Community wisdom and scientific methods were not often at odds, but when they were, honest and open communication was invaluable to balance the two.

## Conclusion

The article outlines the engagement process and protocol development for one community- and patient-engaged study, and in doing so, provides an example of methods that can be used to design and conduct a randomized control trial with a population who has been underrepresented in research and suffered significant historical trauma. Engaging stakeholders in the research process is essential to reduce health inequities [48]. The article is important and relevant because it adds to the current PCOR and engaged research literature and can serve to encourage researchers and funding agencies to be more inclusive of stakeholders in all phases of research.
